# Risk-Stratified Predictive Analysis of Docking Site Outcomes in Lower Extremity Bone Transport: Identifying High-Risk and Low-Risk Zones for Large Segmental Defect Management

**DOI:** 10.3390/jcm15020487

**Published:** 2026-01-08

**Authors:** Gökmen Aktas, Jorge Mayor, Jan Clausen, Ricardo Ramon, Tilman Graulich, Schayan Tabrizi, Maximilian Koblenzer, Hür Özbek, Emmanouil Liodakis, Phillipp Mommsen, Stephan Sehmisch, Tarek Omar Pacha

**Affiliations:** 1 Department of Trauma Surgery, Hannover Medical School, Carl-Neuberg St. 1, 30625 Hannover, Germany; mayorramirez.jorge@mh-hannover.de (J.M.);; 2 Department of Trauma, Hand and Reconstructive Surgery, Departments and Institutes of Surgery, Saarland University, 66123 Homburg, Germany; emmanouil.liodakis@uks.eu

**Keywords:** segmental bone transport, lower extremity bone defects, docking site, Ilizarov, Masquelet

## Abstract

**Background**: Reconstruction of limbs with extensive bone loss often requires complex surgical procedures, which can be technically demanding, time-consuming, and physically and psychologically burdensome for patients. Historically, the lack of alternatives for large bone defects often led to primary amputation. Modern musculoskeletal practice allows for reconstruction using autologous or allogeneic bone grafts, or through more complex procedures such as the Masquelet technique or distraction osteogenesis. However, these methods share a common challenge: the need for a docking site procedure in cases of insufficient bony fusion of the transport segment. The aim of this study was to identify predictive factors for the need for a docking site procedure. **Methods**: A retrospective analysis was conducted on 93 patients treated for lower extremity bone defects between January 2013 and June 2023. Of these, 39 patients (41.9%) underwent segmental bone transport and formed the study cohort for the predictive model analysis. Patients of all ages and both genders were included, regardless of the etiology and size of the defect. The need for a docking site procedure was analyzed using logistic regression, ROC analysis, and ANOVA. **Results**: The study included 93 patients (73 male, 19 female) aged 7 to 83 years. The mean defect size was 76.46 mm (range: 12.1 to 225.1 mm). The mean transport duration was 149.97 days, with a mean transport speed of 0.61 mm/day. Among the 39 segmental transport patients, a docking site procedure was performed in 64.1% (n = 25). Logistic regression and ROC analysis were performed on this subgroup (n = 39, with 25 events). Significant predictors for the need for a docking site procedure were age (*p* = 0.024), vascular injury (*p* = 0.009), transport duration (*p* = 0.001), and transport speed (*p* < 0.001). ROC analysis demonstrated that transport speed (AUC = 0.931) and transport duration (AUC = 0.911) showed strong discriminative ability for predicting docking site procedure necessity, suggesting potential utility as clinical decision-support parameters. **Conclusions**: The study identified transport duration and speed as potentially valuable predictive factors in this retrospective cohort for the need of a docking site procedure, though prospective validation is required. A transport duration exceeding 290.5 days significantly increased the likelihood of requiring a docking site procedure. These findings can help optimize treatment planning and improve long-term limb preservation.

## 1. Introduction

Reconstruction of limbs with extensive bone loss is a significant challenge in modern traumatology [1]. Historically, large bone defects often led to primary amputation due to a lack of viable alternatives [1]. However, advancements in surgical techniques, such as autologous and allogeneic bone grafting, the Masquelet technique, and distraction osteogenesis, have provided new options for limb reconstruction [2,3]. Docking site failure represents a significant clinical burden, occurring in up to 60–70% of bone transport cases and often necessitating additional surgical interventions such as bone grafting, compression osteosynthesis, or revision procedures. Prolonged external fixation increases infection risk, pin-site complications, and patient morbidity, while the unpredictable nature of docking site healing complicates treatment planning and patient counseling. Despite these challenges, validated predictive factors for docking site procedure necessity remain elusive, with existing literature limited by heterogeneous cohorts, inconsistent outcome definitions, and a lack of prospective validation [4,5,6].

Segmental bone transport, a technique pioneered by Gavril A. Ilizarov, has become a standard method for reconstructing large bone defects [7]. This procedure involves the gradual movement of a bone segment to fill a defect, followed by stabilization at the docking site [7]. However, the success of this technique often depends on the ability of the docking site to heal without additional surgical intervention [8,9]. To date, no clear predictive factors for the need for a docking site procedure have been established, leading to decisions based on subjective clinical judgment.

The preliminary study by Omar Pacha et al. provided initial insights into predictive factors for docking site procedures in a cohort of 27 patients with lower extremity bone defects, identifying transport duration as a potential predictor with a cut-off of 188 days (AUC 0.78) [6]. However, the limited sample size precluded robust multivariate analysis, and the cut-off values required validation in larger cohorts. Building upon this foundation, the present study substantially expands the analysis by examining a significantly larger cohort of 93 patients compared to the original 27 patients. This expanded sample allows for a more comprehensive evaluation of both systemic factors (diabetes mellitus, substance abuse, renal insufficiency) and local complications (vascular injuries, wound healing disorders, infection) that might influence docking site healing.

This study aims to identify predictive factors for the need for a docking site procedure in patients undergoing segmental bone transport for large lower extremity bone defects. By analyzing this expanded cohort with more comprehensive parameters, we seek to validate and refine previous findings while providing evidence-based guidelines for optimizing treatment outcomes in clinical practice.

## 2. Materials and Methods

### 2.1. Inclusion and Exclusion Criteria 

A retrospective analysis was conducted on 93 patients treated for lower extremity bone defects between January 2013 and June 2023. Patients of all ages and both genders were included, regardless of the etiology and size of the defect. Patients whose treatment was not yet completed or who discontinued treatment for any reason were excluded. For the predictive model analysis specifically evaluating transport speed, transport duration, and docking site procedure necessity, only the subgroup of patients who underwent segmental bone transport (n = 39) was included. Patients treated with amputation (n = 42) or the Masquelet technique (n = 12) were excluded from this analysis, as transport-specific parameters are not applicable to these treatment modalities. The demographic description (n = 93) includes all patients to provide comprehensive baseline characteristics of our institutional cohort.

### 2.2. Parameters and Definitions

The defect size was measured in millimeters (mm) postoperatively. The duration of transport was defined as the time from the start of transport to the completion of treatment, excluding the time required for the docking site procedure if performed—measured in days. The mean transport speed was calculated as the ratio of transport duration to defect size (mm/day). Radiological analyses were performed using Visage Version 7.1 (Visage Imaging, Berlin, Germany). A docking site procedure was defined as any secondary surgical intervention performed at the docking site to promote union after completion of the transport phase. This included autologous bone grafting, compression osteosynthesis, freshening of bone ends, or any combination thereof. Observation alone without surgical intervention was not classified as a docking site procedure.

Pseudarthrosis was defined as the absence of radiographic union (lack of bridging callus on at least three cortices on orthogonal radiographs) at ≥6 months after completion of bone transport, in conjunction with clinical symptoms (pain, instability) or persistent mobility at the docking site.

The piston technique, as employed in our cohort, combines segmental bone transport using any external fixator system with principles of the induced membrane technique, where a cement spacer is initially placed and subsequently removed during transport to create a biologically favorable membrane environment.

For magnetically driven intramedullary nails, like PRECICE, transport speed was calculated identically to external fixator methods: defect size (mm) divided by transport duration (days), with daily distraction rates programmed according to manufacturer recommendations (typically 0.5–1.0 mm/day) and verified by serial radiographs.

### 2.3. Statistical Analysis

Logistic regression analysis, ROC analysis, and a multivariate analysis of variance (MANOVA) were used to identify predictive factors for the need of a docking site procedure. The cut-off values for each predictive factor were determined using the maximized Youden’s index. Results are presented as standard error of the mean (SEM), standard deviation (SD), odds ratio (OR), and 95% confidence intervals (CI). Prior to logistic regression, multicollinearity was assessed using variance inflation factors (VIF), with VIF > 5 considered indicative of problematic collinearity; no variables exceeded this threshold. All reported *p*-values are two-tailed. Confidence intervals for AUC values were calculated using the default asymptotic normal distribution method implemented in SPSS Version 28 (IBM, Armonk, NY, USA), which assumes approximate normality of the AUC estimator. Missing data were handled using pairwise deletion for descriptive statistics, with available case analysis for each variable. For logistic regression analyses, listwise deletion was applied, including only cases with complete data for all variables in the model. The denominators for each variable are reported in Table 1 to transparently indicate data availability. Missing data were handled using pairwise deletion for descriptive statistics, with available case analysis for each variable. For logistic regression analyses, listwise deletion was applied, including only cases with complete data for all variables in the model.

This research was performed in accordance with the Declaration of Helsinki and has been approved by the local Hannover Medical School Research Ethics Committee. No concerns have been raised (10517_BO_K_2022). Written informed consent was obtained from all patients. In case of minor participants, informed consent was obtained from a parent and/or legal guardian.

## 3. Results

### 3.1. Baseline

The study included 93 patients (73 males, 19 females, one unspecified) with a mean age of 46.13 ± 19.44 years (range: 7–83), BMI of 27.23 ± 6.31 (range: 14.8–45.0), and ASA score of 2.33 ± 0.74 (range: 1–4). Table 1 presents additional patient characteristics, including surgical history (external procedures and revisions), fracture-related variables, healing complications, systemic comorbidities, defect etiology, primary treatment methods, and specific transport techniques employed in the segmental transport group. All data are presented as mean ± standard deviation or frequency (percentage).

### 3.2. Descriptive Analysis

**Figure 1:**
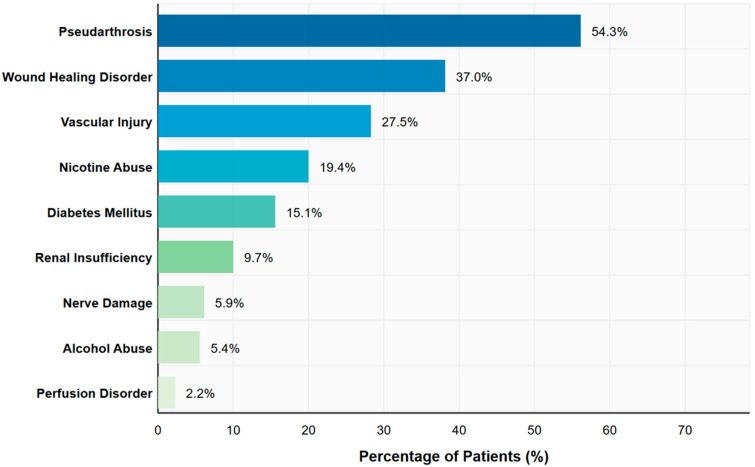
Prevalence of complications and comorbidities in the full study cohort (n = 93) with lower extremity bone defects. This distribution includes all patients regardless of subsequent treatment approach: amputation (45.2%, n = 42), segmental transport (41.9%, n = 39), or Masquelet technique (12.9%, n = 12). Note that transport-specific parameters and predictive analyses apply only to the segmental transport subgroup (n = 39).

Our analysis revealed a high prevalence of complications and comorbidities within the study cohort, as illustrated in Table 1 and Figure 1. Among patients with available data, 86.2% (n = 50) had open fractures. Associated injuries included vascular injuries in 27.5% (n = 14) of patients and nerve damage in 5.9% (n = 3). Perfusion disorders were present in 2.2% (n = 2) of patients, while 9.7% (n = 9) suffered from renal insufficiency.

The observed pseudarthrosis rate of 54.3% exceeds rates commonly reported in the literature (typically 3–30%) (10) and likely reflects the complexity of our tertiary referral cohort, characterized by high rates of open fractures (86.2%), confirmed infections (83.7%), and soft tissue compromise; this is discussed in detail in the Discussion section. Causative microorganisms were detected in 83.7% (n = 77 of 92) of cases (see Table 1). Figure 1 also shows the systemic comorbidities within the study population included diabetes mellitus in 15.1% (n = 14 of 93) of cases, renal insufficiency in 9.7% (n = 9 of 93), and substance use documented with nicotine abuse reported in 19.4% (n = 18 of 93) and alcohol abuse in 5.4% (n = 5 of 93) of patients. External surgical interventions had previously been performed in 39.8% (n = 37 of 93) of patients, with the number of operations at our institution (Hannover Medical School (MHH)) ranging from 1 to 33 per patient (mean 8.37 ± 6.08) and revisions ranging from 0 to 26 (mean 4.59 ± 4.50) (see Table 1). The number of operations per patient ranged from 1 to 33 (mean 8.37 ± 6.08), with 7 operations being the most common (10.8% of patients). Revision surgeries ranged from 0 to 26 (mean 4.59 ± 4.50). No revisions were required in 5.5% (n = 5) of patients, while 19.8% (n = 18) underwent a single revision. Prior external surgical interventions had been performed in 39.8% (n = 37) of patients, with the number of external surgeries ranging from 0 to 8. Most patients (60.2%, n = 56) had no external operations.

**Figure 2:**
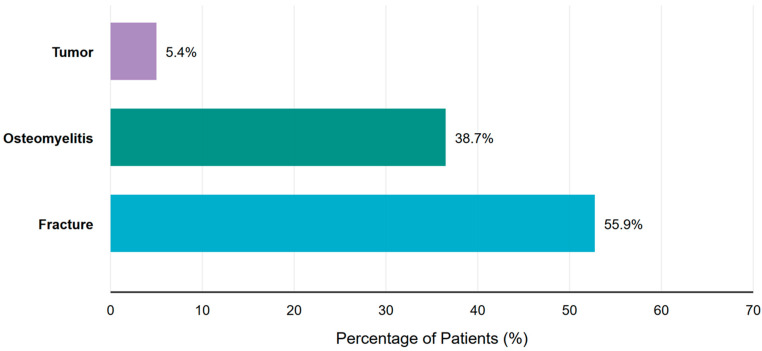
Etiology of bone defects in the total study population (n = 93). This distribution includes all patients irrespective of final treatment modality (amputation, segmental transport, or Masquelet technique). Fractures represent the predominant etiology (55.9%), followed by osteomyelitis (38.7%) and tumors (5.4%).

Regarding the etiology of bone defects, Figure 2 complements Table 1 by demonstrating that fractures were the predominant cause (55.9%), followed by osteomyelitis (38.7%) and tumors (5.4%). This highlights the primarily traumatic and infectious nature of cases in our cohort.

**Figure 3:**
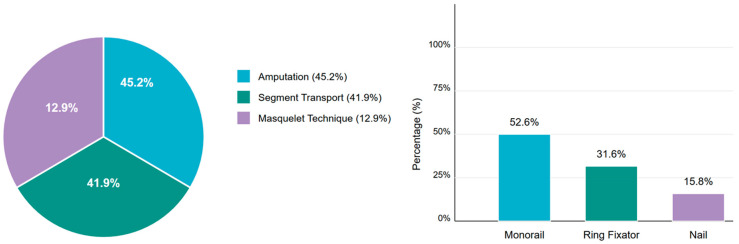
Distribution of treatment approaches and transport methods in patients with lower extremity bone defects. (**Left**) Primary treatment methods used for 93 patients, showing amputation, segmental transport, and the Masquelet technique. (**Right**): Transport methods utilized within the segmental transport group, with the Monorail system, Ring fixator, and intramedullary nail transport.

Complementing the numerical data in Table 1, Figure 3 illustrates the distribution of treatment approaches, revealing comparable utilization of amputation (45.2%, n = 42) and segmental transport (41.9%, n = 39), with the Masquelet technique employed less frequently (12.9%, n = 12). Among segmental transport cases, 53.8% (n = 21) utilized the piston technique. The bar chart component of Figure 3 depicts the transport method distribution, highlighting the Monorail system’s predominance (52.6%, n = 20) over Ring fixator (31.6%, n = 12) and PRECICE nail (15.8%, n = 6) implementations across our clinical cohort.

The mean transport duration, including docking site, was 340.46 ± 202.74 days, with a transport speed of 0.33 ± 0.20 mm/day. Excluding the docking site phase, the mean transport duration was 161.38 ± 101.07 days, with a transport speed of 0.61 ± 0.30 mm/day. A docking site procedure was performed in 64.1% (n = 25) of segmental transport patients.

Antibiotic therapy was administered to 93.5% (n = 86 of 92) of patients. Immunosuppression was rarely used, with 97.8% (n = 90 of 92) receiving no immunosuppressive therapy, 1.1% (n = 1) receiving cortisol, and 1.1% (n = 1) receiving other immunosuppressants.

### 3.3. Inferential Statistics

**Figure 4:**
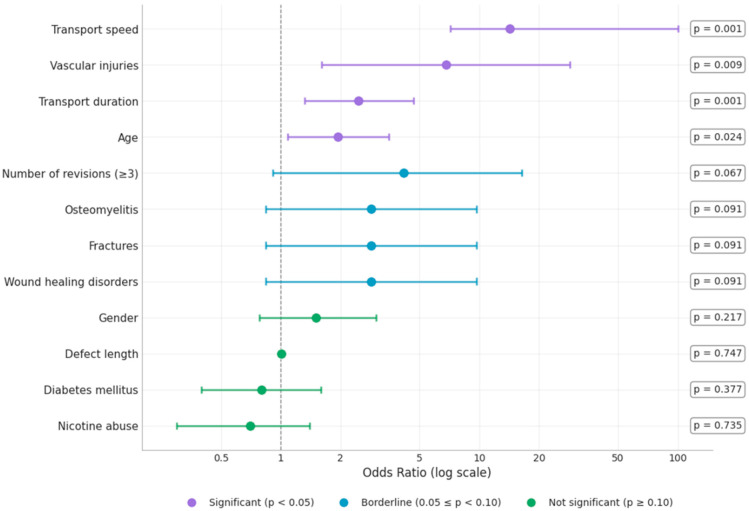
Forest plot of predictors for docking site procedure necessity. Odds ratios (OR) with 95% confidence intervals on logarithmic scale. Purple: significant predictors (*p* < 0.05); blue: borderline significant (0.05 ≤ *p* < 0.10); green: non-significant factors (*p* ≥ 0.10). Vertical dashed line represents OR = 1. Transport speed (OR 14.29), vascular injuries (OR 6.80), transport duration (OR 2.47), and age (OR 1.94) were significant independent risk factors.

Logistic regression identified several significant predictors for docking site procedure necessity: age (*p* = 0.024), vascular injuries (*p* = 0.009), transport duration (*p* = 0.001), and transport speed (*p* < 0.001) (see Table 2, Figure 4). Non-significant variables included gender (*p* = 0.217), defect causing condition (*p* = 0.091), disruption of wound healing (*p* = 0.159), pseudarthrosis (*p* = 0.276), open fracture status (*p* = 0.217), nerve damage (*p* = 0.217), renal insufficiency (*p* = 0.217), causative microbes (*p* = 0.217), diabetes mellitus (*p* = 0.377), BMI (*p* = 0.405), ASA score (*p* = 0.310), nicotine abuse (*p* = 0.735), and alcohol abuse (*p* = 0.377) (see Table 2, Figure 4).

ROC curve analysis showed high predictive value for transport speed (AUC = 0.931, 95% CI: 0.857–1.000) and transport duration (AUC = 0.911, 95% CI: 0.806–1.000). Defect length showed no significant predictive value (AUC = 0.531, 95% CI: 0.343–0.720, *p* = 0.747).

**Figure 5:**
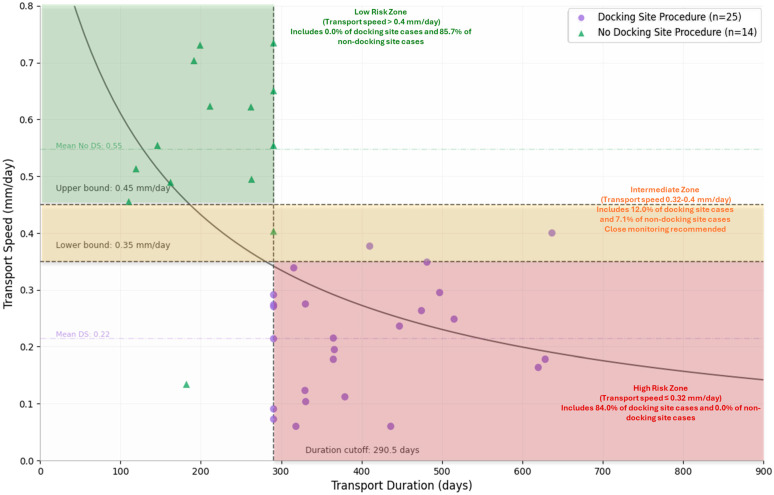
Risk stratification matrix correlating (four-quadrant plot) transport speed (mm/day) versus duration (days) with docking site procedure outcomes: purple circles (n = 25) required docking site procedures, green triangles (n = 14) did not. Cutoff thresholds (vertical: 290.5 days; horizontal: 0.35–0.4 mm/day) and risk zones demonstrate clear separation between outcome groups, confirming high predictive value of these parameters (AUC: 0.931 for speed, 0.911 for duration).

The optimal cut-off for transport duration was 290.5 days (sensitivity 72%, specificity 85.7%), and for transport speed 0.134 mm/day (sensitivity 72%, specificity 100%) (see Figure 5). It should be noted that the mean transport speed of 0.61 ± 0.30 mm/day represents the observed average across all segmental transport patients, while the ROC-derived cut-off of 0.134 mm/day represents the threshold that maximizes discrimination between patients requiring and not requiring docking site procedures. This cut-off falls at the extreme lower end of the distribution and identifies patients with particularly slow transport progression who are at the highest risk. The ROC-derived optimal cut-off of 0.134 mm/day represents the statistical threshold maximizing Youden’s index, while the clinically oriented risk zones (0.35–0.4 mm/day) were established based on visual inspection of the scatter plot distribution to identify practically meaningful boundaries that separate outcome groups. The 0.134 mm/day cut-off falls within the high-risk zone and represents an extreme value; the broader zone boundaries (0.35–0.4 mm/day) were selected to provide clinically applicable thresholds that account for measurement variability and enable prospective patient stratification.

MANOVA comparison of transport durations across the different surgical treatment methods showed no statistically significant differences (*p* = 0.329), despite numerical differences: Ring fixator (403.58 days, 95% CI: 256.53–550.63), PRECICE nail (362.17 days, 95% CI: 93.46–630.87), and Monorail system (292.85 days, 95% CI: 213.60–372.10). The effect size was eta-squared = 0.062 (95% CI: 0.000–0.220).

Post hoc Bonferroni tests confirmed no significant differences between transport methods: Monorail versus Ring fixator (mean difference −110.73 days, *p* = 0.440), Monorail versus Nail (mean difference −69.32 days, *p* = 1.000), and Ring fixator versus Nail (mean difference 41.42 days, *p* = 1.000).

Figure 4 presents a comprehensive forest plot visualizing the predictive factors for docking site procedure necessity using odds ratios (OR) with corresponding 95% confidence intervals on a logarithmic scale. The vertical dashed line represents an OR of 1.0, which indicates no effect. Points to the right of this line suggest increased risk, while points to the left suggest decreased risk. The forest plot categorizes predictors by statistical significance using color coding: significant predictors (*p* < 0.05) in blue, borderline significant (0.05 ≤ *p* < 0.10) in orange, and non-significant factors (*p* ≥ 0.10) in red. Four significant independent risk factors are prominently displayed: transport speed (OR 14.29, 95% CI: 2.80–71.43, *p* < 0.001) emerged as the strongest predictor, followed by vascular injuries (OR 6.80, 95% CI: 1.47–31.48, *p* = 0.009), transport duration (OR 2.47, 95% CI: 1.42–4.29, *p* = 0.001), and age (OR 1.94, 95% CI: 1.09–3.45, *p* = 0.024). Borderline significant factors include defect-causing condition (*p* = 0.091) and disruption of wound healing (*p* = 0.091), both with ORs suggesting potential influence but not reaching statistical significance. The remaining factors show no significant predictive value (*p* ≥ 0.10), with confidence intervals crossing the line of no effect (OR = 1.0). Notably, traditional risk factors for impaired healing, such as diabetes mellitus (OR 0.47, *p* = 0.377), nicotine abuse (OR 1.25, *p* = 0.735), and alcohol abuse (OR 0.47, *p* = 0.377), did not demonstrate significant associations with docking site procedure necessity.

Figure 5 illustrates the relationship between transport speed and duration in determining the need for docking site procedures, showing a clear visual separation between patients who required procedures (purple circles, n = 25) and those who did not (green triangles, n = 14). The plot is divided into clinically relevant risk zones by a vertical line at 290.5 days (transport duration) and horizontal lines at 0.35 mm/day and 0.4 mm/day (transport speed). This reveals three distinct zones: a Low Risk Zone (transport speed > 0.4 mm/day) containing 85.7% of non-docking site cases and no docking site cases; an Intermediate Zone (transport speed 0.35–0.4 mm/day) including 12.0% of docking site cases and 7.1% of non-docking site cases where close monitoring is recommended; and a High Risk Zone (transport speed ≤ 0.35 mm/day) encompassing the vast majority of docking site cases (84.0%) with no non-docking site cases. The ROC-derived optimal cut-off of 0.134 mm/day represents the statistical threshold maximizing Youden’s index, while the clinically oriented risk zones (0.35–0.4 mm/day) were established based on visual inspection of the scatter plot distribution to identify practically meaningful boundaries that separate outcome groups. The 0.134 mm/day cut-off falls within the high-risk zone and represents an extreme value; the broader zone boundaries (0.35–0.4 mm/day) were selected to provide clinically applicable thresholds that account for measurement variability and enable prospective patient stratification. The plot demonstrates remarkable predictive accuracy (transport speed AUC = 0.931; duration AUC = 0.911), confirming that all patients with transport duration > 290.5 days AND speed < 0.35 mm/day required docking site procedures, while most patients with faster speeds (>0.4 mm/day) did not require intervention.

## 4. Discussion

Building upon our previous study “Predictive Factors for docking site procedure in bone transport for large lower extremity segmental defects,” we have now examined 93 patients with various types of segmental transport for bone defects of the lower extremities and comorbidities with respect to predictive factors for the necessity of a docking site procedure [1]. In addition to these factors, Risk Zones for the necessity of a docking site operation were created based on our data. The most reliable predictors were transport duration with a valid cut-off value above 290.5 days and transport speed with a cut-off value of 0.1340 mm/day.

The four-quadrant representation in Figure 5, formed by the identified cut-off values for transport speed (horizontal: 0.35–0.4 mm/day threshold zone) and transport duration (vertical: 290.5 days), demonstrates a potential prognostic stratification approach based on our empirical findings. The lower right quadrant (speed < 0.35 mm/day, duration > 290.5 days) constitutes a high-risk zone where all 7 patients (100%) required a docking site procedure, yielding a positive predictive value (PPV) of 100% for this zone. The upper left quadrant (speed > 0.4 mm/day, duration < 290.5 days) represents a low-risk zone containing 12 of 14 patients (85.7%) who did not require docking site procedures and none of the 25 patients who required procedures, corresponding to a negative predictive value (NPV) of 100% within this zone. The overall model sensitivity for identifying patients requiring docking site procedures was 72.0% (18/25 patients correctly identified by the duration threshold > 290.5 days), while the specificity was 85.7% (12/14 patients correctly classified as not requiring intervention). It should be noted that the intermediate zone (speed 0.35–0.4 mm/day) contains patients from both outcome groups, highlighting the need for close clinical monitoring in this range. These predictive values should be interpreted cautiously, given the small sample sizes in individual quadrants, and require prospective validation.

This risk stratification model represents a methodologically innovative approach to assessing docking site procedure likelihood by integrating two readily obtainable clinical parameters. Despite the significant correlations observed, our findings are constrained by the limited sample size (n = 39 segmental transport patients, of which n = 25 required docking site procedures). The model could potentially serve as a foundation for future refined frameworks that incorporate additional parameters such as vascularity, soft tissue coverage, and specific defect locations—factors identified as potentially significant in both our analysis and existing literature [2]. Such developments could contribute substantially to standardizing assessment and treatment protocols for docking site complications in segmental transport cases, though prospective validation in larger cohorts remains essential before clinical implementation can be recommended.

Our results differ from our previous findings, “Predictive factors for docking site procedure in bone transport for large lower extremity segmental defects”. While a cut-off value of 188 days for transport duration was identified there (sensitivity 72%, specificity 67%, AUC 0.78), the updated data with 93 patients show a cut-off value at 290.5 days (sensitivity 72%, specificity 85.7%, AUC 0.911) [1]. This discrepancy in cut-off values likely reflects fundamental differences in cohort composition. The pilot study (n = 27) exclusively included patients who underwent segmental bone transport, whereas the current analysis encompasses a broader institutional cohort of 93 patients with various treatment outcomes, of which only 39 (41.9%) received segmental transport. Additionally, as a tertiary referral center, our expanded cohort may include proportionally more complex cases with longer treatment durations, potentially explaining the shift toward higher cut-off thresholds. The improved diagnostic accuracy (AUC 0.911 vs. 0.78) in the current study may reflect the larger sample size, providing more stable estimates.

Particularly striking is the difference in transport speed as a predictor. While a cut-off at 0.32 mm/day with moderate predictive power (sensitivity 78%, specificity 44%, AUC 0.48) was found in our previous study, the results of this study show a significantly lower cut-off of 0.1340 mm/day with better diagnostic accuracy (sensitivity 72%, specificity 100%, AUC 0.931). This discrepancy could be due to the different transport methods [1].

Our study included 93 patients, predominantly male (78.5%), with a higher prevalence of tibial defects (73.1%) compared to femoral defects (26.9%). The main causes of the defects were fractures (55.9%), osteomyelitis (38.7%), and tumors (5.4%), with a remarkably high rate of open fractures (86.2% of documented cases). Despite the obvious gender imbalance in our cohort, gender did not prove to be a significant predictor (*p* = 0.217) for complications or the necessity of intervention, in contrast to some studies suggesting gender-specific differences in bone healing and complication rates.

Regarding treatment approaches, amputation was the most commonly applied treatment in our cohort (45.2%), followed by segmental transport (41.9%) and the Masquelet technique (12.9%). Among the 39 patients who received segmental transport, 53.8% used the piston technique, which we included in our previous study, resembling a combination of a classic external segmental transport by the LRS monorail system and the first step of the Masquelet technique, which the literature recently reported as the piston technique. Retrospectively, the reasons for amputation could not be reconstructed, for example, using the Mangled Extremity Severity Score (MESS). Beyond amputations, transport methods showed variations between Monorail (52.6%), Ring fixator (31.6%), and intramedullary nail transport (15.8%). The relatively low use of nail transport (15.8%) is consistent with observations that bone transport with magnetic nails is a relatively new technique with limited evidence, mainly from case series and case reports [3,10].

Docking site procedures were required in 64.1% of segmental transport cases. Our results are consistent with a systematic analysis by Liodakis et al., which showed that planned docking site interventions significantly improved union rates [4]. In their pooled analysis of 1153 patients from 23 studies, 90% of docking sites with planned interventions achieved union without further intervention, compared to only 66% in pure observation groups (*p* < 0.0001) [4].

Our data analysis showed high rates of pseudarthrosis (54.3%) and impaired wound healing (37.0%), highlighting the complexity in treating bone defects. Microbial pathogens were detected in 83.7% of patients, emphasizing the infectious component in many cases.

Surprisingly, comorbidities such as diabetes mellitus (15.1%, *p* = 0.377), nicotine abuse (19.4%, *p* = 0.735), and alcohol abuse (5.4%, *p* = 0.377) did not prove to be significant predictors for pseudarthrosis or the need for additional interventions despite their prevalence in our cohort [4]. Upon closer examination of our Forest Plot (Figure 4), some comorbidities show point estimates below 1 (e.g., diabetes mellitus or nicotine abuse OR of 0.47, *p* = 0.377 and OR 1.25, *p* = 0.735). However, these findings should not be interpreted as protective effects. The wide 95% confidence intervals for all these factors cross the neutral line at OR = 1, indicating that the observed point estimates are statistically indistinguishable from no effect. The apparent direction of these non-significant associations likely reflects random variation in our limited sample rather than true biological relationships. These unexpected results contradict conventional clinical expectations that these comorbidities would significantly influence healing outcomes [4]. Similar results were reported by Liodakis et al. in their systematic review, in which patient-specific factors showed inconsistent associations with docking site union rates [4]. Given our limited sample size (n = 93), however, caution is advised in interpreting these negative results, as the study may lack the statistical power to detect smaller effect sizes associated with these comorbidities.

The high prevalence of pseudarthrosis in our cohort (54.3%) significantly exceeds the rates reported in some studies, such as by Feng et al., who found pseudarthrosis at the docking site in only 3.9% of cases among 103 patients with tibial defects treated with bone transport [2]. This significant discrepancy could be explained by the particularly complex nature of our cases, including the high rate of open fractures (86.2% of available cases) and confirmed infections (83.7%). Additionally, methodological differences in the definition and diagnosis of pseudarthrosis may contribute to this variance [2]. These divergent results underscore the need for standardized reporting of complications in the literature on bone transport.

Our results suggest that anatomical and mechanical factors may be more predictive than patient-specific comorbidities, providing a differentiated perspective that contributes to the existing evidence base [1,2,5]. Feng et al. conducted a more comprehensive analysis of risk factors in 103 patients with tibial defects and found that “the blood supply status is related to the quality of osteotylus formation at the docking site,” specifically identifying the distal third location (OR: 11.379) and soft tissue defects as independent risk factors for delayed healing. Their multivariate analysis showed that bone defect length was also a significant predictor, with an OR of 1.976, suggesting that each additional centimeter of the defect nearly doubled the risk of delayed healing [2]. Similarly, they found that the external fixation time (EFT) had an OR of 1.017, indicating a cumulative risk with longer fixation [2].

The high rate of tibial injuries (73.1%) in our cohort could partially explain the frequency of pseudarthrosis, as the limited vascularity of the distal tibia presents challenges for bone healing [2,5]. This anatomical consideration is consistent with the findings of Feng et al. that “bone defects in the distal third and soft tissue defects are independent risk factors for delayed healing” due to the fewer arteries supplying nutrients in this region [2]. Our data on the frequency of vascular injuries (27.5%) and wound healing disorders (37.0%) provide additional context for understanding the vascular challenges in these cases. In comparison, Zhong Wan Run reported a pseudarthrosis rate of 28.6% at the docking site in patients with soft tissue defects, which is closely aligned with our results, despite our smaller sample size [2]. This convergence of results across different studies strengthens the evidence for soft tissue status as a predictive factor, despite the limitations of our study.

In our logistic regression analysis, we identified significant predictors for docking site procedures, including age (*p* = 0.024), vascular injury (*p* = 0.009), transport duration in days (*p* = 0.001), and transport speed (*p* < 0.001) (see Table 2). The ROC analysis showed that transport speed has a high diagnostic accuracy (AUC = 0.931) for predicting docking site procedures, while transport duration also showed high predictive power (AUC = 0.911). These findings provide valuable insights for clinical decision-making regarding the planning of docking site interventions.

Interestingly, our MANOVA and post hoc tests showed no significant differences in transport duration between different transport methods (Monorail, Ring fixator, PRECICE nail) (*p* = 0.329), with mean transport durations of 292.85 days for Monorail, 403.58 days for Ring fixator, and 362.17 days for PRECICE nail systems. This finding suggests that, although the technologies differ, the overall treatment duration remains similar across different methods. From a clinical perspective, our findings suggest several practical applications pending prospective validation. First, transport speed can be monitored during treatment; patients demonstrating speeds below 0.35 mm/day may benefit from early counseling regarding the high likelihood of requiring docking site procedures and potentially from proactive surgical planning. Second, the risk stratification matrix could facilitate shared decision-making by providing patients with individualized risk estimates based on readily measurable parameters. Third, for patients falling within the high-risk zone, clinicians might consider earlier planned docking site interventions rather than awaiting docking site failure, potentially reducing overall treatment duration and external fixation time. However, we emphasize that these clinical applications require prospective validation before implementation. It should be noted that transport duration and external fixation time (EFT) are inherently correlated parameters, as reported by Feng et al., who identified EFT as an independent risk factor (OR 1.017 per day). Our finding that longer transport duration predicts docking site procedure necessity may partially reflect the cumulative biological effects of prolonged external fixation, including pin-site colonization, soft tissue tethering, and bone quality deterioration. Future studies should attempt to disentangle these interrelated temporal factors.

Antibiotics were administered to 93.5% of patients, reflecting the high prevalence of infections in our cohort. Fung et al. identified tibial defects and larger defect sizes as significant risk factors for postoperative infections [6]. Hsu et al. found that initially infected pseudarthrosis and defect lengths over seven centimeters were risk factors for post-infections when using the induced membrane procedure [7].

Patients in our study underwent an average of 8.37 ± 6.08 operations (range: 1–33) and 4.59 ± 4.50 revisions (range: 0–26), with external interventions performed in 39.8% of patients. This quantifies the considerable surgical burden these patients experience and underscores the importance of developing effective prediction models to minimize unnecessary interventions while ensuring optimal outcomes [7,8,9,11]. Comparable studies on the frequency of external and subsequent in-house revisions are not known to us to date.

Our sample size (n = 93, with only 39 patients who received segmental transport) limits the statistical power for identifying predictive factors. A particular statistical consideration is the risk of overfitting in our multivariate logistic regression model [12,13]. With 25 events (docking site procedures) and four significant predictors identified, our events-per-variable ratio of approximately 6:1 falls below the commonly recommended threshold of 10:1 to 15:1 for stable regression estimates [12,13]. This limitation is reflected in the wide confidence intervals observed, particularly for transport speed (OR 14.29, 95% CI: 2.80–71.43), indicating estimation instability due to limited sample size. The precision of our odds ratio estimates should therefore be interpreted with appropriate caution. While the identified predictors demonstrate statistically significant associations, the exact magnitude of effect sizes may vary in larger validation cohorts. External validation in independent, adequately powered datasets is essential before these findings can be reliably implemented in clinical decision-making algorithms [12,13]. For comparison, Feng et al. included 103 patients specifically with tibial defects, while Liodakis et al. meta-analyzed 1153 patients [11,14]. This smaller sample increases the risk of both Type I and Type II errors in the analysis of predictive factors.

The retrospective design of our study also leads to inherent biases in data collection and analysis, potentially affecting the reliability of certain results. Heterogeneity among patients represents another limitation. Our cohort included various defect etiologies (fractures, osteomyelitis, tumors), different anatomical locations (although predominantly tibia), and a wide range of defect sizes (12.1 mm to 225.1 mm). This heterogeneity, while reflecting real-life clinical practice, could obscure findings that would be apparent in more homogeneous populations.

The lack of standardized definitions for key parameters such as pseudarthrosis and wound healing complications in the literature makes direct comparison with other studies challenging. Our remarkably high pseudarthrosis rate (54.3%) compared to other published series (e.g., 3.9% reported by Feng et al.) highlights this definitional inconsistency in the field [15].

Furthermore, our study did not include patient-reported outcome measures or quality of life assessments, which would provide valuable insights into the functional and psychological impacts of these complex procedures, the duration of therapy, and their complications. Such data would help contextualize the clinical significance of docking site procedures beyond purely surgical endpoints.

Finally, although we have identified significant predictors for the necessity of a docking site procedure, our model does not account for surgeon-specific decision-making factors that might influence the threshold for intervention. The decision to perform a docking site procedure involves clinical judgment that may vary between surgeons and institutions, potentially biasing our outcome measure.

## 5. Limitations

Our study has several important limitations that should be considered when interpreting the results. The retrospective design introduces inherent biases in data collection and analysis, potentially affecting the reliability of certain findings.

The sample size, particularly within the segmental transport subgroup (n = 39), limits statistical power for identifying predictive factors, especially when compared to larger studies in the literature. This relatively small sample increases the risk of both Type I errors and Type II errors when analyzing potential predictors. The reduced power is particularly relevant for detecting more subtle associations, potentially explaining why established risk factors like diabetes and smoking did not emerge as significant predictors in our analysis. Furthermore, with four predictors and only 25 events, our multivariate model carries substantial overfitting risk, as evidenced by the wide confidence intervals (e.g., transport speed OR 14.29, 95% CI: 2.80–71.43). These estimates should be considered exploratory pending external validation.

Patient heterogeneity represents another limitation. Our cohort included various defect etiologies (fractures, osteomyelitis, tumors), different anatomical locations (though predominantly tibia), and a wide range of defect sizes (12.1 mm to 225.1 mm). This heterogeneity, while reflective of real-world clinical practice, may obscure findings that would be apparent in more homogeneous populations. The inclusion of different transport methods (Monorail, ring fixator, intramedullary nail) further compounds this variability, although our analysis found no significant differences between these approaches.

The lack of standardized definitions for key parameters such as pseudarthrosis and wound healing complications across the literature makes direct comparison with other studies challenging. Our notably high pseudarthrosis rate (54.3%) compared to other published series (e.g., 3.9% reported by Feng et al.) highlights this definitional inconsistency in the field (10).

Additionally, our study did not include patient-reported outcome measures or quality-of-life assessments, which would provide valuable insights into the functional and psychological impact of these complex procedures and their complications. Such data would help contextualize the clinical significance of docking site procedures beyond mere surgical endpoints.

Finally, while we identified significant predictors for docking site procedure necessity, our model does not account for surgeon-specific decision-making factors that might influence the threshold for intervention. The decision to perform a docking site procedure involves clinical judgment that may vary between surgeons and institutions, potentially introducing bias in our outcome measure. Additionally, the retrospective nature of our study precluded blinded outcome assessment, potentially introducing observer bias in the determination of pseudarthrosis and the decision to perform docking site procedures. Surgeon-dependent variability in intervention thresholds represents another limitation; some surgeons may have lower thresholds for performing docking site procedures, which could influence our outcome rates. Furthermore, we did not systematically record soft tissue quality using standardized classifications such as the Gustilo-Anderson grading system for open fractures. Given the established importance of soft tissue status in bone transport outcomes, the absence of this parameter limits our ability to fully characterize wound-related risk factors and may represent an important unmeasured confounder.

These limitations highlight the need for larger, prospective multicenter studies with standardized definitions, assessment protocols, and outcome measures to further validate our findings and develop more robust predictive models for optimizing treatment approaches in this challenging patient population.

## 6. Conclusions

Our study provides valuable insights into the management of bone defects, with particular focus on segmental transport and docking site procedures. The identification of transport speed and duration as significant predictors for docking site procedure necessity offers clinicians practical guidance for treatment planning. Notably, the development of clearly defined risk zones enables evidence-based decision-making, with clear separation between high-risk patients (100% requiring docking site procedures with low speed and long duration) and low-risk patients, providing practical guidance for early intervention decisions.

The surprisingly limited influence of traditional risk factors such as diabetes and smoking suggests that anatomical and mechanical factors may play a more dominant role in determining outcomes than previously recognized. Given the high rates of pseudarthrosis (54.3%) and docking site procedures (64.1% of segmental transport cases), targeted strategies for improving docking site union warrant further investigation.

The considerable surgical burden documented in our analysis (average 8.37 operations per patient) underscores the need for strategies to reduce interventions while maintaining or improving outcomes. The development of predictive models for high surgical burden could facilitate more efficient treatment planning and resource allocation.

Despite limitations in sample size and study design, our findings contribute to the growing evidence base guiding complex bone defect management. Future studies should employ prospective designs with standardized outcome measures, including patient-reported outcomes and quality of life assessments. Future predictive models should incorporate objective measures of bone and soft tissue quality, vascular status, and infection parameters to better stratify risks. The relatively low proportion of nail transport (15.8%) in our cohort presents an opportunity for investigating newer techniques such as magnetic nail transport, which may offer advantages in selected cases [10]. Prospective, multicenter validation is required to standardize risk thresholds for transport speed and duration before clinical implementation can be recommended.

## Figures and Tables

**Table 1:** Overview of demographic characteristics, clinical factors, surgical history, systemic comorbidities, defect etiology, primary treatment methods, and specific transport techniques. MHH = Hannover Medical School; ASA = American Society of Anesthesiologists physical status classification.

Characteristics	Value/Frequency	Range/Percent
Demographic Factors		
Sex		
- Male	n = 73	78.5%
- Female	n = 19	20.4%
- Unspecified	n = 1	1.1%
Age	46.13 ± 19.44 years	7–83 years
BMI	27.23 ± 6.31	14.8–45.0
ASA score	2.33 ± 0.74	1–4
Fracture-Associated Factors		
Open fractures	n = 50 of 58	86.2%
Vascular injuries	n = 14 of 51	27.5%
Nerve damage	n = 3 of 51	5.9%
Surgical History		
External surgeries performed	n = 37 of 93	86.2%
Number of operations (MHH)	8.37 ± 6.08	1–33
Number of revisions (MHH)	4.59 ± 4.50	0–26
Healing Complications		
Pseudarthrosis	n = 50 of 92	54.3%
Disruption of wound healing	n = 34 of 92	37.0%
Perfusion disorders	n = 2 of 93	2.2%
Detection of causative microbes	n = 77 of 92	83.7%
Systematic Comorbidities		
Diabetes mellitus	n = 14 of 93	15.1%
Nicotine abuse	n = 18 of 93	19.4%
Alcohol abuse	n = 5 of 93	5.4%
Renal insufficiency	n = 9 of 93	9.7%
Etiology of Bone Defects		
Fractures	n = 52 of 93	55.9%
Osteomyelitis	n = 36 of 93	38.7%
Tumors	n = 5 of 93	5.4%
Treatment Methods		
Amputation	n = 42 of 93	45.2%
Monorail	n = 20 of 38	52.6%
Ring fixator	n = 12 of 38	31.6%
Intermedullary nail	n = 6 of 38	15.8%
Docking site procedure performed	n = 25 of 39	64.1%

**Table 2:** Overview of factors analyzed for docking site procedure necessity. Parameters organized by significance level with corresponding frequency/value data, *p*-values, area under curve (AUC) measurements where applicable, and clinical significance. Statistical significance was defined as *p* < 0.05 for highly significant predictors, 0.05 ≤ *p* < 0.10 for borderline significant factors, and *p* ≥ 0.10 for non-significant parameters.

Parameter	Frequency/Value	*p*-Value	AUC (95% CI)	Clinical Significance
Highly Significant Predictors				
Transport speed	0.61 ± 0.30 mm/day	*p* < 0.001	0.931 (0.857–1.000)	Highest predictive value for docking site surgery
Transport duration	340.46 ± 202.5 days	*p* = 0.001	0.911 (0.806–1.000)	Longer duration increases probability
Vascular injuries	27.5% of cases	*p* = 0.009	-	Significant risk factor
Age	46.13 ± 19.44 years	*p* = 0.024	-	Significant demographic factor
Defect Causes and Associated Factors				
Fractures	55.9% of all cases	*p* = 0.091	-	Most common cause, borderline significant
- Open fractures	86.2% of fracture cases	*p* = 0.217	-	High complication rate, but not predictive
Osteomyelitis	38.7% of all cases	*p* = 0.091	-	Second most common cause, often chronic
- Microbiological evidence	83.7% of cases	-	-	High detection rate, 93.5% antibiotic therapy
Tumors	5.4% of all cases	-	-	Too few cases for statistical analysis
Complications and Associated Factors				
Wound healing disorders	37.6% of all cases	*p* = 0.091	-	Borderline significant predictor
Pseudarthrosis	54.8% of all cases	*p* = 0.276	-	Common, but not predictive
Nerve damage	5.9% of all cases	*p* = 0.217	-	Rare complication, not predictive
Treatment Strategies				
Transport methods	Monorail: 52.6% Ring fixator: 31.6% Nail: 15.8%	*p* = 0.329	-	No method superiority demonstrable
Docking site surgery rate	64.1% of all transports	-	-	High intervention rate
Non-significant Demographic/Anatomical factors				
Gender	M: 78.5%, F: 20.4%	*p* = 0.217	-	No influence
Defect length	78.99 ± 45.45 mm	*p* = 0.747	0.531 (0.343–0.720)	No predictive value
Affected bone	Femur: 26.9% Tibia: 73.1%	*p* = 0.735	-	No influence of location
Comorbidities				-
Renal insufficiency	9.7%	*p* = 0.217	-	No influence
Diabetes mellitus	14.0%	*p* = 0.377	-	No influence
Nicotine abuse	20.4%	*p* = 0.735	-	No influence
Alcohol abuse	5.4%	*p* = 0.377	-	No influence

## Data Availability

The datasets supporting the conclusions of this article are fully presented within the manuscript. Further underlying datasets, including raw data, can be made available by the corresponding author upon reasonable request.
